# Tyrosinase inhibitory activity, molecular docking studies and antioxidant potential of chemotypes of *Lippia origanoides* (Verbenaceae) essential oils

**DOI:** 10.1371/journal.pone.0175598

**Published:** 2017-05-01

**Authors:** Alessandra P. da Silva, Natália de F. Silva, Eloísa Helena A. Andrade, Tais Gratieri, William N. Setzer, José Guilherme S. Maia, Joyce Kelly R. da Silva

**Affiliations:** 1 Programa de Pós-Graduação em Biotecnologia, Universidade Federal do Pará, Belém, Pará, Brazil; 2 Programa de Pós-Graduação em Ciências Farmacêuticas, Universidade Federal do Pará, Belém, Pará, Brazil; 3 Coordenação de Botânica, Museu Paraense Emílio Goeldi, Belém, Pará, Brazil; 4 Laboratory of Food, Drug and Cosmetics (LTMAC), School of Health Sciences, University of Brasilia, Brasília, Distrito Federal, Brazil; 5 Department of Chemistry, University of Alabama in Huntsville, Huntsville, Alabama, United States of America; 6 Programa de Pós-Graduação em Recursos Naturais da Amazônia, Universidade Federal do Oeste do Pará, Santarém, Pará, Brazil; Islamic Azad University Mashhad Branch, ISLAMIC REPUBLIC OF IRAN

## Abstract

The essential oils (EOs) of the aerial parts of *Lippia origanoides* (**LiOr**), collected in different localities of the Amazon region, were obtained by hydrodistillation and analyzed by GC and CG-MS. Principle component analysis (PCA) based on chemical composition grouped the oils in four chemotypes rich in mono- and sesquiterpenoids. Group I was characterized by 1,8-cineole and α-terpineol (**LiOr-1** and **LiOr-4**) and group II by thymol (**LiOr-2**). The oil **LiOr-3** showed β-caryophyllene, α-phellandrene and β-phellandrene as predominant and **LiOr-5** was rich in (*E*)-nerolidol and β-caryophyllene. All samples were evaluated for antioxidant activity and inhibition of tyrosinase *in vitro* and *in silico*. The highest antioxidant activity by the DPPH free radical method was observed in **LiOr-2** and **LiOr-5** oils (132.1 and 82.7 mg TE∙mL^-1^, respectively). The tyrosinase inhibition potential was performed using L-tyrosine and L-DOPA as substrates and all samples were more effective in the first step of oxidation. The inhibition by samples **LiOr-2** and **LiOr-4** were 84.7% and 62.6%, respectively. The samples **LiOr-1**, **LiOr-4** and **LiOr-5** displayed an interaction with copper (II) ion with bathochromic shift around 15 nm. In order to elucidate the mechanism of inhibition of the main compounds, a molecular docking study was carried out. All compounds displayed an interaction between an oxygen and Cu or histidine residues with distances less than 4 Å. The best docking energies were observed with thymol and (*E*)-nerolidol (-79.8 kcal.mol^-1^), which suggested H-bonding interactions with Met281 and His263 (thymol) and His259, His263 ((*E*)-nerolidol).

## Introduction

The enzyme tyrosinase (EC 1.14.18.1; catechol oxidase, monophenol monooxygenase, monophenol, dihydroxyphenylalanine: oxygen oxidoreductase, polyphenol oxidase) is a metalloenzyme group of polyphenol oxidases that occurs in various organisms and perform some specific functions in melanogenesis [1]. The substrates L-tyrosine and L-DOPA are involved in the first and second steps of melanin biosynthesis, respectively [2]. Firstly, the tyrosinase promotes the transformation of L-tyrosine into L-DOPA by hydroxylation and then L-DOPA is converted to dopaquinone (diphenolase activity is the second step of tyrosinase reaction) [3]. Furthermore, tyrosinase catalyzes the oxidation of 5,6-di-hydroxyindole (DHI) to form indole-5,6-quinone of the melanin precursors [4]. Because tyrosinase participates in at least three stages of melanogenesis, tyrosinase inhibition would serve to block melanin biosynthesis and would be important as a base of formulations used for the treatment of skin blemishes [5].

A large number of moderate to potent tyrosinase inhibitors from natural and synthetic resources have been reported during the last decade [6,7]. Tyrosinase inhibitors such as arbutin, azelaic acid, electron-rich phenols, hydroquinones and kojic acid have been tested in pharmaceuticals and cosmetics for their capability of preventing overproduction of melanin [8]. However, arbutin and kojic acid have shown little inhibitory activity against pigmentation in intact melanocytes in a clinical trial, and hydroquinone is considered cytotoxic to melanocytes and potentially mutagenic to mammalian cells [9]. Therefore, it remains necessary to search for new tyrosinase inhibitors from botanical sources without side effects. The traditional use of plants against skin diseases, especially for cosmetic purposes is a common practice in the folk medicine of many cultures and new discoveries have provided better depigmenting agents [10].

The genus *Lippia* (Verbenaceae) is well-known for its aromatic character and comprises around 200 species of herbs, shrubs and small trees spread distributed in South and Central America and Tropical Africa [11,12]. *Lippia origanoides* Kunth (syn. *Lippia berteroi* Spreng, *Lippia schomburgkiana* Schauer) is an aromatic shrub up to 3 m in height, known popularly as “alecrim d’angola” and “salva-do-marajó”, growing wild in savanna areas of North Brazil [13]. Many studies have reported its biological activities such as antimicrobial [14], anti-hypertensive [15], antispasmodic, anti-inflammatory, analgesic [16] and antioxidant [17]. In addition, its essential oil (EO) presents a number of substances with antioxidant activity such as thymol, carvacrol and 1,8-cineole, which act as captors of free radicals, thus stressing the importance in combating damage from reactive oxygen species leading to premature aging [18,19].

Considering the wealth of the Amazon biodiversity and the need to promote sustainable use, this study aims at the discovery of bioactive compounds present in essential oils in species native to the Amazon, antioxidant and tyrosinase inhibitory potential. From a commercial point of view, these essential oils have not yet been explored and if applied in new cosmetic formulations can attract consumers with a preference for natural cosmetics and add economic value to important species in the region.

## Material and methods

### Chemicals

Tyrosinase from mushroom (MuTyr) (T3824), Trizma (T5941), Tween 20 (P9416), DMSO (Dimethyl sulfoxide) (D8418), DPPH (2,2-Diphenyl-1-picrylhydrazyl; C_18_H_12_N_5_O_6_) (D9132), kojic acid (C_6_H_6_O_4_) (220469), L-dihydroxyphenylalanine (L- DOPA, D9628), and L-tyrosine (T3754) were purchased from Sigma (St. Louis, MO, USA). Potassium phosphate monobasic (KH_2_PO_4_) and potassium phosphate dibasic (Na_2_HPO_4_∙ 2H_2_O) were obtained from VETEC (Rio de Janeiro, RJ, Brazil). Trolox^®^ (6-hydroxy-2,5,7,8-tetramethylchroman-2-carboxylic acid) was purchased from ACROS ORGANICS. All solvents used (*n*-hexane, ethanol, and methanol) were of analytical grade and purchased from TEDIA (Fairfield, OH, USA).

### Plant material

The plant material was collected in different localities from Pará and Maranhão states (Brazil) during the rainy season (Table 1). The plant material collects in all collection site was authorized by Instituto Chico Mendes da Conservação da Biodiversidade (Chico Mendes Institute for Biodiversity Conservation, Brazil). We confirm that the field studies did not involve endangered or protected species. The vouchers were deposited in the herbarium of Emílio Goeldi Museum, city of Belém, Pará state, Brazil for cataloging and botanical identification.

**Table 1:** Samples of *Lippia origanoides* collected in the Amazon region.

Cod.	Voucher	Code	Locality	Geographic coordinates
PPB 123	MG180326	**LiOr-1**	Anapurus, MA	3° 40′ 19″ S, 43°6′ 57″ W
PPB328	MG 33921	**LiOr-2**	Parauapebas, PA	6° 6′ 29″ S, 50°18′ 16″ W
PPN009	MG200147	**LiOr-3**	São Geraldo do Araguaia, PA	6° 24′ 3″ S, 48°33′ 18″ W
PPN058	MG200170	**LiOr-4**	Mirador, MA	6° 22′ 15″ S, 44°21′ 46″ W
PPN107	NR*	**LiOr-5**	Parauapebas, PA	6° 6′ 29″ S, 50°18′ 16″ W

*NR: not registered. The botanical identification was made by comparison with authentic samples.

### Plant processing

The aerial parts of plants (leaves and twigs) were air-dried and pulverized. The EOs were obtained by hydrodistillation using a Clevenger-type apparatus (100 g, 3 h). The oils were dried over anhydrous sodium sulfate, and their percentage contents were calculated on the basis of the dry weight of plant material.

### Oil composition analysis

The quantitative analysis was performed by gas chromatography with flame ionization detector (Focus GC-FID, Thermo Scientific^™^). Sample solutions in hexane (2 μL/1000 μL) were prepared and 1.0 μL were injected under the following conditions: silica capillary column DB-5 MS (30 m × 0.25 mm × 0.25 μm), carrier gas: nitrogen (flow rate: 1 2 mL/min) injection mode split (20:1) temperature of column 60 to 240°C (range of 3°C/min) and injector and detector temperatures 250°C. For qualitative analysis of the essential oils gas chromatography-mass spectrometry (DSQ II GC-MS, Thermo Scientific^™^) was used. The analysis conditions for the injector and column were the same for GC-FID. The ionization source is electron impact (70 eV); transfer line temperature 200°C, helium carrier gas. The structural identification was made by comparison of their mass spectra and retention index to existing data in system libraries NIST 2011 and Adams 2007 [20,21].

### Free-radical scavenging

A solution of DPPH radical 0.5 mM was prepared in methanol with initial absorbance of approximately 0.625 ± 0.02. Each oil (5 μL) was mixed with 900 μL of 100 mM Tris-HCl buffer (pH 7.4), 10 μL of Tween 20 0.5% (w/w) and then was added 1.0 mL of DPPH^•^ (250 μM in the reaction mixture) [22]. The mixture was mixed vigorously for 1 minute and maintained in the dark at room temperature. The absorbance of the samples was measured at 517 nm by UV-Visible (Biosystems spectrometer) in continuous intervals of 30 minutes for a duration of two hours. For the negative control, the samples were replaced by methanol and the percentage inhibition of DPPH^•^ was calculated by Eq 1. Trolox, a water-soluble equivalent of vitamin E, was used as positive control [23]. The percentage inhibitions of the oils were compared with the inhibition induced by 1 mM of Trolox solution. The total antioxidant capacity (TEAC) was expressed to mg ET∙mL^-1^ of oil.

$$I_{DPPH}\left(\%\right)=\left(\frac{A_{c}-A_{a}}{A_{c}}\right)\times 100$$ (1)

Where:

Ac = Absorbance of negative control at 517 nm

Aa = absorbance of the sample at 517 nm

### Tyrosinase bioassay

The inhibition of tyrosinase was determined by a modification of the dopachrome method using L-DOPA and L-tyrosine as substrate [24]. The samples were dissolved in DMSO at initial concentration of 20 mg∙mL^-1^ and then diluted in phosphate buffer (pH 6.8) at concentration of 1.0 mg∙mL^- 1^. In a cuvette, 400 μL of each sample were placed and tyrosinase solution at (0.1 mg∙mL^- 1^) in 800 μL phosphate buffer (0.1M, pH 6.8) was added. Then 400 μL of the substrate (L-tyrosine or L-DOPA) (0.5 mg∙ml^-1^) was added and incubated for 30 minutes at 37°C. After 30 min of reaction, the absorbance was read at 492 nm and the inhibition percentage calculated in relation to the control. Phosphate buffer and kojic acid were tested under the same conditions as negative and positive control, respectively.

### Chelation capacity of copper ions

A UV-visible spectral curve (250–500 nm) was constructed to determine the power chelation of Cu^2+^ [25]. A copper (II) sulfate solution (250 μM) was used as source of Cu^2+^ ions. All reactions consisted in 1 mL of copper II sulfate to 1 mL of samples. (1.0 mg∙mL^-1^). The UV-visible spectrum was obtained after 10 minutes of incubation at 25°C.

### Molecular docking

In order to assess the interaction between the major compounds present in the more active essential oils with tyrosinase, a molecular docking analysis was carried out using the program *Molegro Virtual Docker* (MVD) [26]. The 3-D structure of mushroom tyrosinase complexed with the inhibitor tropolone was obtained from the Protein Data Bank (PDB code 2Y9X) [27]. MVD used in this docking study calculates a MolDock Score (*E*_*MolDock*_), which is defined sum of intermolecular, *E*_*inter*_ (the ligand–enzyme interaction energy) and intramolecular energy *E*_*intra*_ (the internal energy of the ligand) terms:

$$E_{MolDock}=E_{inter}+E_{intra}$$

and The *E*_*inter*_ is determined by the following:

$$E_{inter}=\sum_{i=ligand}\sum_{j=enzyme}[E_{PLP}\left(r_{ij}\right)+\;332,0\;\frac{q_{i}q_{j}}{4r_{ij}^{2}}]$$

The *E*_*PLP*_ term is a piecewise linear potential (PLP) using two different parameters: one parameter approximates the steric (van der Waals interactions) term between atoms and another Coulombic potential for hydrogen bonds. The PLP describes others interaction types, such as repulsive, buried, nonpolar, H-bonding and metals [26]. Metals are treated as “heavy atoms” with the appropriate charge (+2 in the case of copper). Electrostatic interactions are Coulomb potentials and include a steric clash penalty for distances < 2.0 Å.

The *E*_*intra*_ is calculated by the following:

$$E_{intra}=\;\sum_{i=ligand}\sum_{j=enzyme}\left[E_{PPL}\left(r_{ij}\right)\right]+\;\sum_{flexible\;bonds}A\left[1-\text{cos }\left(m\theta -\theta_{0}\right)\right]+\;E_{clash}$$

The double summation term calculates all the energies between atom paris of the ligand, excluding atom pairs connected by two bonds or fewer. The second term is a torsional energy term, where *θ* is the torsional angle of the bond. The average of the torsional energy bond contribution is used if several torsions have been determined. The last term *E*_*clash*_ assigns a penalty of 1000 if the distance between two atoms (more than two bonds apart) is less than 2.0 Å, serving to punish unrealistic ligand conformations [26].

### Statistical analysis

Samples were assayed in triplicate in the DPPH and tyrosinase assays. The results are shown as means ± standard deviation and analysis of variance was conducted by Tukey test (P <0.05) using GraphPad Prism 5.0 software. All volatile compounds identified were used as variables in the Principal Component Analysis and a matrix of correlation was applied and two components (PC1 and PC2) were computed. These data were analyzed using the XLSTAT software (free version).

## Results and discussion

### Chemical composition of essential oils and PCA analysis

Eighty-four volatile components were identified, comprising a range of 86.8 to 100.0% of the total composition of the oils (Table 2). The EOs from *L*. *origanoides* showed different volatile profiles among the samples. The oils of **LiOr-1**, **LiOr-2** and **LiOr-4** were dominated by oxygenated monoterpenoids (81.4–90.2%). For the oil **LiOr-3**, the most representative classes were monoterpene hydrocarbons (35.3%) and sesquiterpene hydrocarbons (34.6%); the oil **LiOr-5** displayed the higher concentration of oxygenated sesquiterpenoids (35.8%) and oxygenated monoterpenoids (24.4%).

**Table 2:** Chemical composition of *Lippia origanoides* oils in relative percentage (%).

Constituent	RI_Calc_	RI_Lit._	LiOr-1	LiOr-2	LiOr-3	LiOr-4	LiOr-5
α-Thujene	916	924		0.2			
α-Pinene	934	932	2.5	0.3		4.6	3.1
Sabinene	976	969	2.2			4.9	
β-Pinene	985	974	1.0	0.1		2.5	
α-Phellandrene	1006	1002	0.2	0.1	17.6		0.5
δ-3-Carene	1008	1008				0.5	0.3
α-Terpinene	1014	1014	0.4			0.7	
*p*-Cymene	1027	1020	0.9	**6.4**			0.7
β-Phellandrene	1027	1025			17.7		
1,8-Cineole	1031	1026	**64.1**			**70.5**	**7.7**
(*Z*)-β-Ocimene	1034	1032	0.1	1.1			
γ-Terpinene	1054	1054	3.7			1.7	2.2
*cis*-Sabinene hydrate	1066	1065				1.1	
*cis*-Linalool oxide (furanoid)	1067	1067					0.1
Terpinolene	1081	1086	0.3	0.1		0.5	
Linalool	1096	1095		0.5	4.2		1.2
*trans*-Sabinene hydrate	1098	1098	0.5			1.2	
*cis*-*p*-menth-2-en-1-ol	1122	1136	0.2				
α-Campholenal	1123	1122					0.2
*trans*-Pinocarveole	1135	1135	0.2				0.4
*trans*-Limonene oxide	1137	1137	0.1				
*cis*-Verbenol	1145	1137					0.3
Pinocarvone	1157	1160					0.2
δ-Terpineol	1165	1162	0.6			0.5	
Umbellulone	1166	1167		0.1			
*p*-Menth-1,5-dien-8-ol	1167	1166					0.8
Terpinen-4-ol	1175	1174	3.0	0.3	0.5	3.1	1.0
*p*-Cymen-8-ol	1182	1179					0.1
α-Terpineol	1190	1186	**12.0**			**5.0**	2.7
*trans*-Carveol	1213	1215					0.1
Thymol methyl ether	1217	1232	0.7	0.1			1.9
Thymol	1279	1289	1.1	88.2			2.4
Carvacrol	1285	1298		1.0			**5.3**
δ-Elemene	1335	1335	0.1				
α-Cubebene	1347	1345					2.5
α-Copaene	1368	1374	0.3	0.1	0.7	0.4	
β-Elemene	1381	1389				0.2	
7-*epi*-Sesquithujene	1380	1390			0.3		
Sesquithujene	1395	1405			0.4		
α-*cis*-Bergamotene	1406	1411			0.4		
β-Caryophyllene	1411	1417	3.8	0.3	22.1	1.0	**12.7**
β-Gurjunene	1420	1431			0.4		
β-Copaene	1421	1430					0.1
α-*trans*-Bergamotene	1425	1432	0.1		0.2		
α-Guaiene	1429	1437					0.1
(*Z*)-β-Farnesene	1433	1440			0.8		
α-Humulene	1445	1452	0.2	0.7		0.3	1.7
Geranyl acetone	1440	1453			0.6		
Sesquisabinene	1449	1457			0.4		
(*E*)-β-Farnesene	1457	1454	0.2				
γ-Muurolene	1466	1478	0.1		0.4		
Amorpha-4,7(11)-diene	1472	1479			0.3		
γ-Curcumene	1470	1481			0.8		
Germacrene D	1484	1484	0.1				
Bicyclogermacrene	1498	1500	0.1				
α-Muurolene	1489	1500	0.1		0.2		
*trans*-Muurola-4(14),5-diene	1509	1493					2.2
δ-Amorphene	1500	1511		0.1			
β-Curcumene	1501	1514			1.8		
*trans*-Calamenene	1515	1521			4.3		
δ-Cadinene	1508	1522	0.2		1.1		
(*E*)-γ-Bisabolene	1529	1529	0.1				
Sesquisabinene hydrate	1534	1542			0.2		
(*E*)-Nerolidol	1553	1561			0.5		**27.9**
Spathulenol	1577	1577	0.1				
Caryophyllene oxide	1571	1582	0.5		4.9		3.2
Globulol	1583	1590			1.7		3.0
Humulene epoxide II	1597	1608			0.4		
1,10-di-*epi*-Cubenol	1615	1618			0.2		
1-*epi*-Cubenol	1616	1627			0.2		
α-Acorenol	1621	1632			0.9		
Caryophyllne-4(12),8(13)-dien-5α-ol	1625	1639			1.0		1.2
*allo*-Aromadendrene epoxide	1632	1639	0.1				0.3
α-Muurolool	1636	1644					0.2
Caryophylla-4(14),8(15)-dien-5α-ol	1639	1639	0.1				
α-Cadinol	1643	1652			1.0		
Selin-11-en-4α-ol	1646	1658			0.3		
*epi*-β-Bisabolol	1660	1670			0.7		
14-Hydroxy-9-*epi*-(*E*)-caryophyllene	1668	1668	0.1				
α-Bisabolol	1675	1685			2.1		
(2*E*,6*Z*)-Farnesal	1699	1713			1.3		
(2*E*,6*E*)-Farnesal	1727	1740			2.0		
Isoamyl (*E*)-cinnamate	1741	1740			0.5		
Monoterpene hydrocarbons			11.3	8.3	35.3	15.4	6.8
Oxygenated monoterpenoids			82.5	90.2	4.7	81.4	24.4
Sesquiterpene hydrocarbons			5.4	1.2	34.6	1.9	19.3
Oxygenated Sesquiterpene			0.9		18.0		35.8
Others					0.5		
**Total identified**			**100.0**	**99.7**	**93.1**	**98.7**	**86.8**

RI^Calc.^ = based on DB-5ms capillary column and alkane Standards (C8-C32). RI^Lit.^ = based on the book of Adams (2007).

Bold letters representing compounds above than 5%

**Fig 1:**
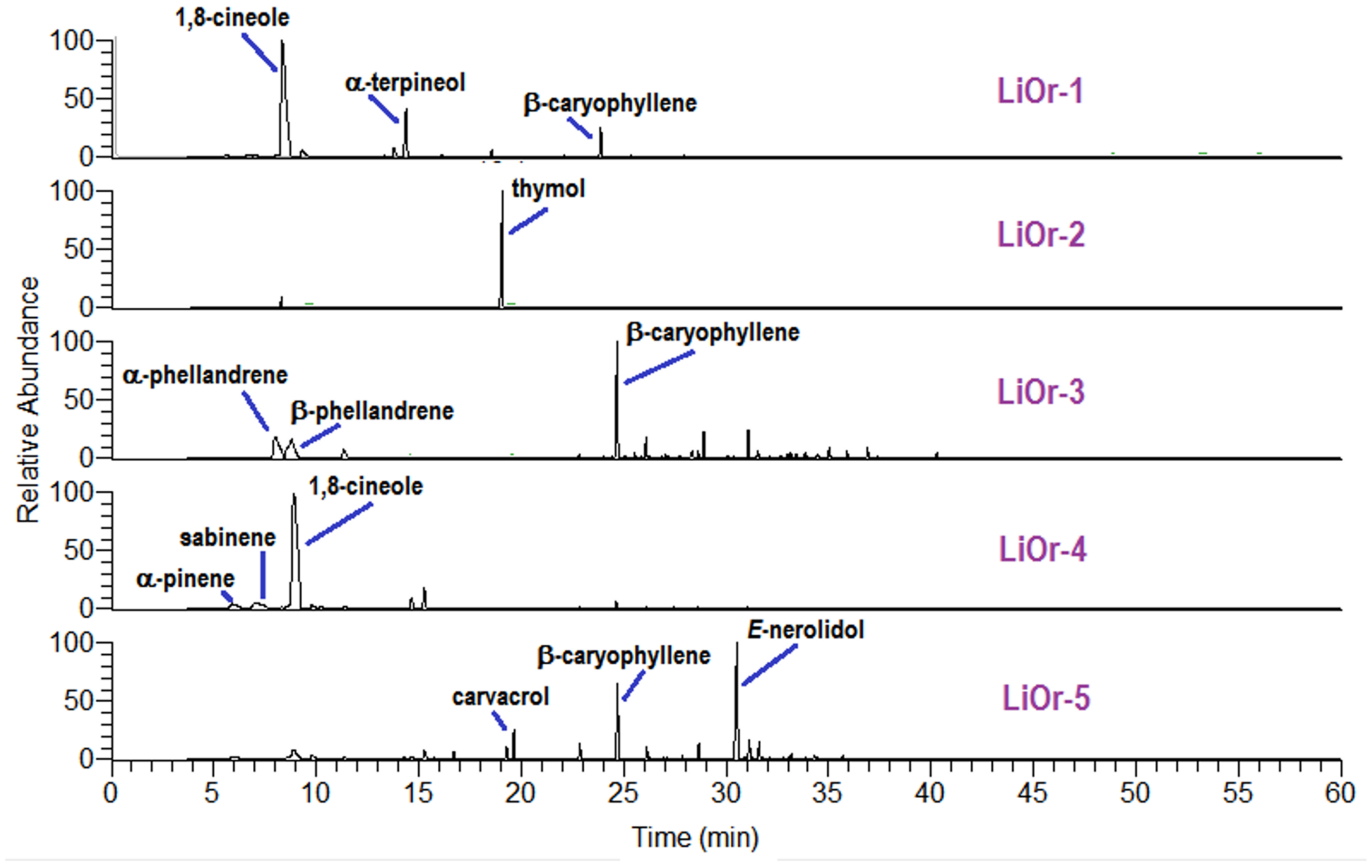
Chemical profile obtained for *Lippia origanoides* oils by GC-MS analysis.

The main compounds identified in the **LiOr-1** oil were 1,8-cineole (64.1%) followed by α-terpineol (12.0%) and β-caryophyllene (3.8%). The oxygenated monoterpene 1,8-cineole (70.5%) was also the major compound in the **LiOr-4** oil, followed by α-pinene (4.6%) and sabinene (4.9%). The **LiOr-2** oil was dominated by thymol (88.2%) and smaller amounts of *p*-cymene (6.4%), (*Z*)-β-ocimene (1.1%) and carvacrol (1.0%). β-Caryophyllene (22.1%), β-phellandrene (17.7%) and α-phellandrene (17.6%) were predominant in the **LiOr-3** oil and the sample **LiOr-5** was rich in (*E*)-nerolidol (27.9%), β-caryophyllene (12.7%) and carvacrol (5.3%). The chemical profiles of the samples are shown in Fig 1.

**Fig 2:**
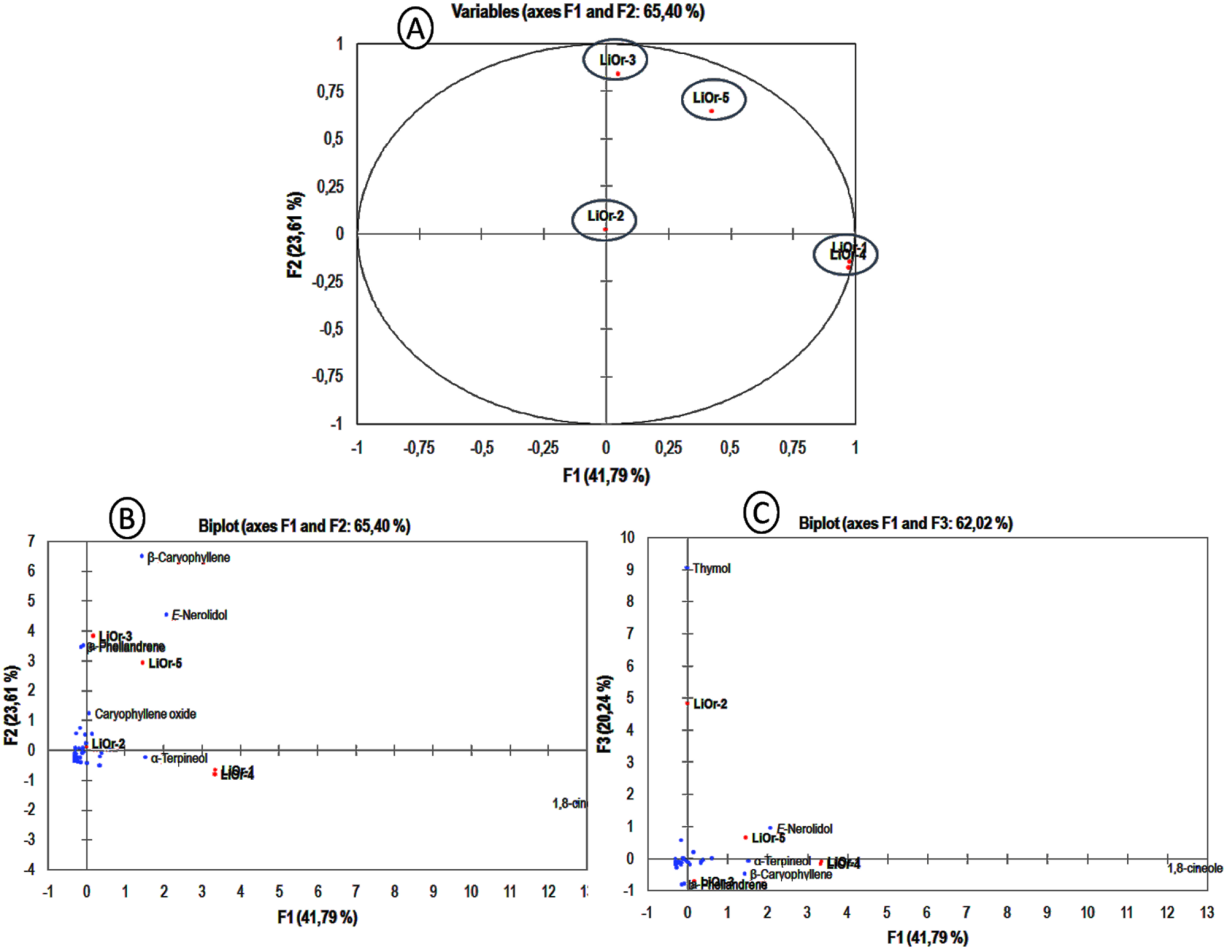
PCA analysis of *L*. *origanoides* based on EO chemical composition. (A) Bidimensional plot of first two components (PC1 and PC2). (B) Variables contributions to components PC1 and PC2. (C) Variables contributions to components PC2 and PC3.

The compounds identified in the all samples were used as variables in the PCA analysis and the samples were classified into 4 groups. The components PC1, PC2 and PC3 have accounted for 41.79%, 23.61% and 20.24% of phytochemical variability, respectively. PC1 had positive correlation of 1,8-cineole (91.6%), (*E*)-nerolidol (2.4%), α-terpineol (1.3%) and β-caryophyllene (1.2%). The samples **LiOr-1** and **LiOr-4** which presented 1,8-cineole (64.1 and 70.1%, respectively) as main compound displayed the more positives loadings (0.96 and 0.95). PC2 with 23.61% of variance showed positive correlation with β-caryophyllene (42.1%), (*E*)-nerolidol (20.7%), α-phellandrene (12.3%) and β-phellandrene (11.9%). The oils **LiOr-3** and **LiOr-5**, which presented the higher amounts of β-caryophyllene and (*E*)-nerolidol displayed in the graph the more positive loadings (0.71 and 0.42, respectively). The component PC3 had a positive influence of thymol and it is represented by **LiOr-2** oil. The different levels of contribution of the compounds can be observed in Fig 2.

The occurrence of different chemical profiles in *L*. *origanoides* EOs have been reported and these include monoterpene hydrocarbons, oxygenated monoterpenes and sesquiterpene hydrocarbons as main compounds. The oils collected in four different regions of Colombia (Santander, Cauca, Nariño and Boyacá) were classified in three chemotypes: A, B and C. The chemotype A was characterized by the presence of *p*-cymene (12%), β-caryophyllene (9%), α-phellandrene (8%), β-phellandrene (6%), limonene (5%), α-humulene (5%) and 1,8-cineole (ca. 4%). The chemotypes B and C were dominated by carvacrol (ca. 40%) and thymol (56%), respectively, followed by *p*-cymene (9–13%) and γ-terpinene (5–11%) [28]. In addition, the seasonal and circadian studies of EOs of *L*. *origanoides* displayed a higher variation of methyl (*E*)-cinnamate, (*E*)-nerolidol, *p*-cymene, 1,8-cineole, carvacrol, α-pinene, β-caryophyllene and γ-terpinene throughout the year and it was the first report of a methyl (*E*)-cinnamate / (*E*)-nerolidol chemotype [29].

### Antioxidant activity of essential oils by DPPH method

The percentage inhibition for EO samples were calculated and expressed as Trolox equivalents as shown in Table 3. An inhibition curve was plotted based on Trolox concentrations of 0.25, 0.50, 0.75,1.0 and 1.5 mM and the range of inhibition values obtained were 15.8 to 86.8%. The line equation was obtained by linear regression (y = 0.017x—0.051, R^2^ = 0.998) and was used to express the results in mg of Trolox equivalents per mL of oil (mg ET∙mL^-1^).

**Table 3:** DPPH scavenging activity of *Lippia origanoides* oils.

Sample	Inhibition (%)	mg ET∙mL^-1^
**LiOr-1**	27.6 ± 12.0	43.8 ± 21.3
**LiOr-2**	77.5 ± 0.3	132.1 ±0.6
**LiOr-3**	25.1 ± 2.4	39.2 ± 4.3
**LiOr-4**	16.5 ± 1.5	24.1 ± 2.7
**LiOr-5**	49.6 ± 0.2	82.7 ± 0.3

The higher antioxidant activity was observed to sample **LiOr-2** (132.1 mg ET∙mL^-1^), which is rich in thymol. Thymol is reported as one of the natural products more active in protecting the quality of food and bodies for damage induced by oxidative stress [30,31]. Phenolic compounds commonly present an antioxidant or pro-oxidant activity according to their concentration [32]. The presence of a phenolic ring in the structure promotes antioxidant activity due to the ability to scavenge free radicals, donation of hydrogen atoms or electrons, or complexation of metal ions [33].

The sample **LiOr-5** displayed a moderate activity (82.7 mg ET∙mL^-1^) and it was rich in sesquiterpenes such as (*E*)-nerolidol (27.9%) and β-caryophyllene (12.7%). The isomer (*Z)*-nerolidol was able to scavenge DPPH and hydroxyl radicals it in a dose-dependent manner [34]. EOs rich in caryophyllene have been reported as natural antioxidants. The EO of *Licaria rigida*, dominated by β-caryophyllene (76.1%) showed a high antioxidant activity in the DPPH assay (718.1 mg TE∙mL^-1^) [35]. In addition, caryophyllene displayed a strong antioxidant activity, with IC_50_ values of 1.25 and 3.23 μM equivalents of ascorbic acid in DPPH and FRAP methods, respectively [36].

**LiOr-1** and **LiOr-4** showed antioxidant activity of 39.2 and 24.1 mg ET∙mL^-1^, respectively. The oxygenated monoterpenoid 1,8-cineole was the major compound in both, however, **LiOr-1** showed a significant amount of α-terpineol (12.0%) and terpinen-4-ol (4.0%). The antioxidant activity of these compounds has been reported in different systems. The EO of *Ocimum basilicum*, rich in 1,8-cineole (31.2%), exhibited high radical-scavenging activity of both DPPH and ABTS radicals [37]. In Addition, the pure compound 1,8-cineole was subjected to DPPH and β-carotene bleaching assays and displayed remarkable activities in both systems [38]. The DPPH radical-scavenging capacity was determined for α-terpineol and terpinen-4-ol, which showed activities only 2 times weaker than Trolox [23]. The difference in radical scavenging between **LiOr-1** and **LiOr-4** may be attributed to synergism in the antioxidant activity between the compounds present in these oils.

### Inhibition of tyrosinase activity

**Fig 3:**
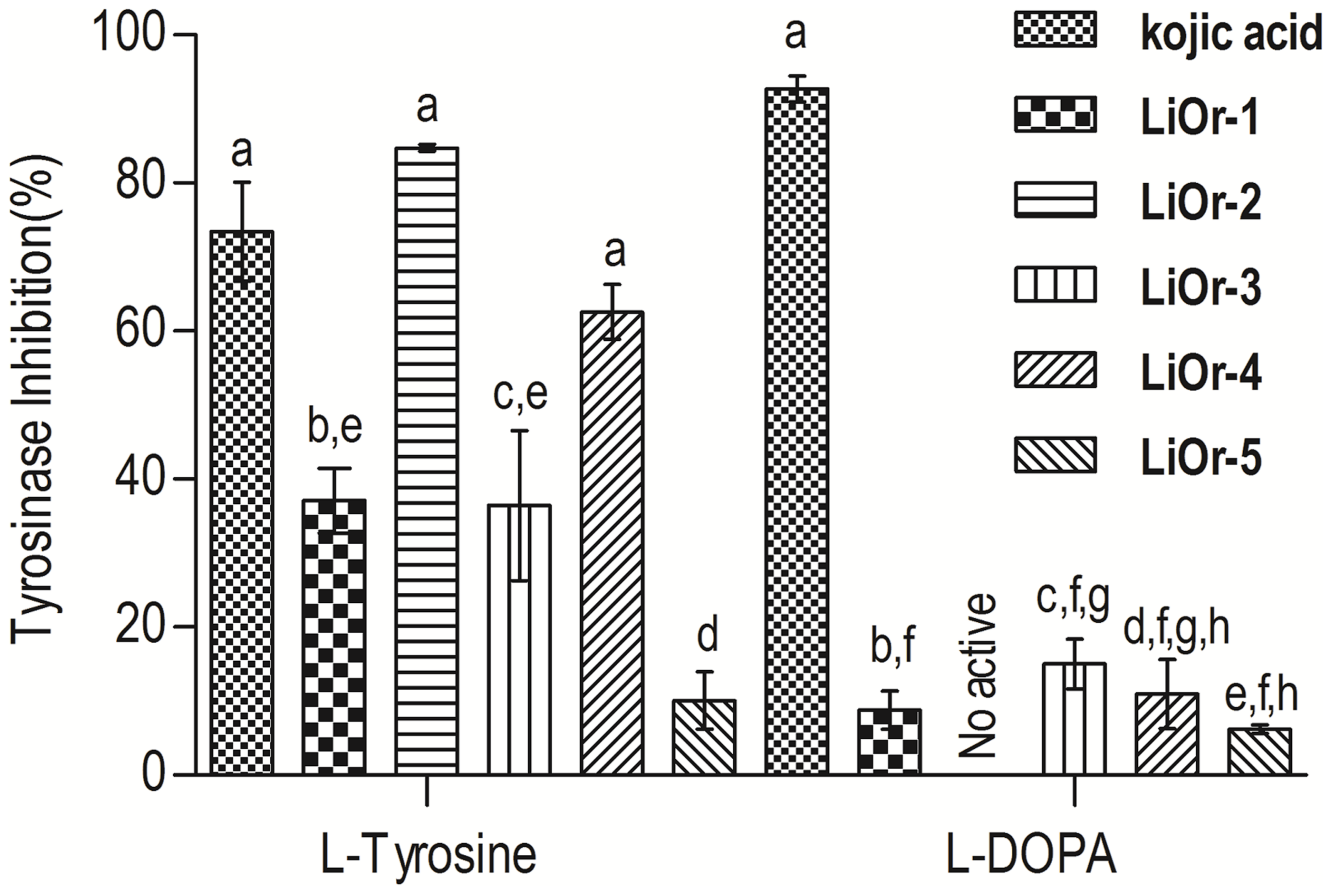
Tyrosinase inhibitory activity for *Lippia origanoides* essential oils. ^a,b,c,d,e,f,g,h^ Values with different letters are statistically different at the *p* < 0.05 level (Tukey’s test).

The samples were tested using two substrates, L-tyrosine and L-DOPA, and the samples showed a remarkable and weak inhibition, respectively (Fig 3). In the reaction of L-tyrosine the samples **LiOr-2** and **LiOr-4** showed higher inhibition values (> 50.0%). However, the samples did not display activity with L-DOPA as the substrate.

The oil **LiOr-2**, rich in thymol (88.2%), caused 84.7% of tyrosinase inhibition. Recently, a new inhibitory mechanism of thymol to dopachrome formation from mushroom tyrosinase has been proposed by Satooka and Kubo (2011). *N*-Acetyl-L-tyrosine was used as substrate and it suggested that thymol inhibits redox chemical reactions of the dopaquinone leuko-dopachrome instead of the enzymatic reaction. The redox inhibitory activity of thymol was evaluated for a redox reaction with L-DOPA and *p*-benzoquinone. Thymol successfully inhibited L-DOPA to dopaquinone oxidation, coupled with the reduction of *p*-benzoquinone. Thus, the suppression of the formation of dopachrome by thymol is due to inhibition of leuko-dopachrome to dopachrome conversion. The antioxidant properties of thymol are considered as a key feature to the mechanism of inhibition of melanin synthesis [39].

The oils **LiOr-1** and **LiOr-4** were rich in 1,8-cineole and α-terpineol and displayed an inhibition of 37.1% and 62.6% to the substrate L-tyrosine. The EO of *Eucalyptus camaldulensis* (Myrtaceae) presenting 1,8-cineole (23.9%) and γ-terpinene (13.9%), α-eudesmol (11.6%) and γ-eudesmol (8.0%) at concentration of 5.2 mg/mL inhibited 82.9% of mushroom tyrosinase activity. In addition, this oil inhibited intracellular tyrosinase activity and then decreased the melanin content in B16F10 cells [40].

### Interaction effect of copper

**Fig 4:**
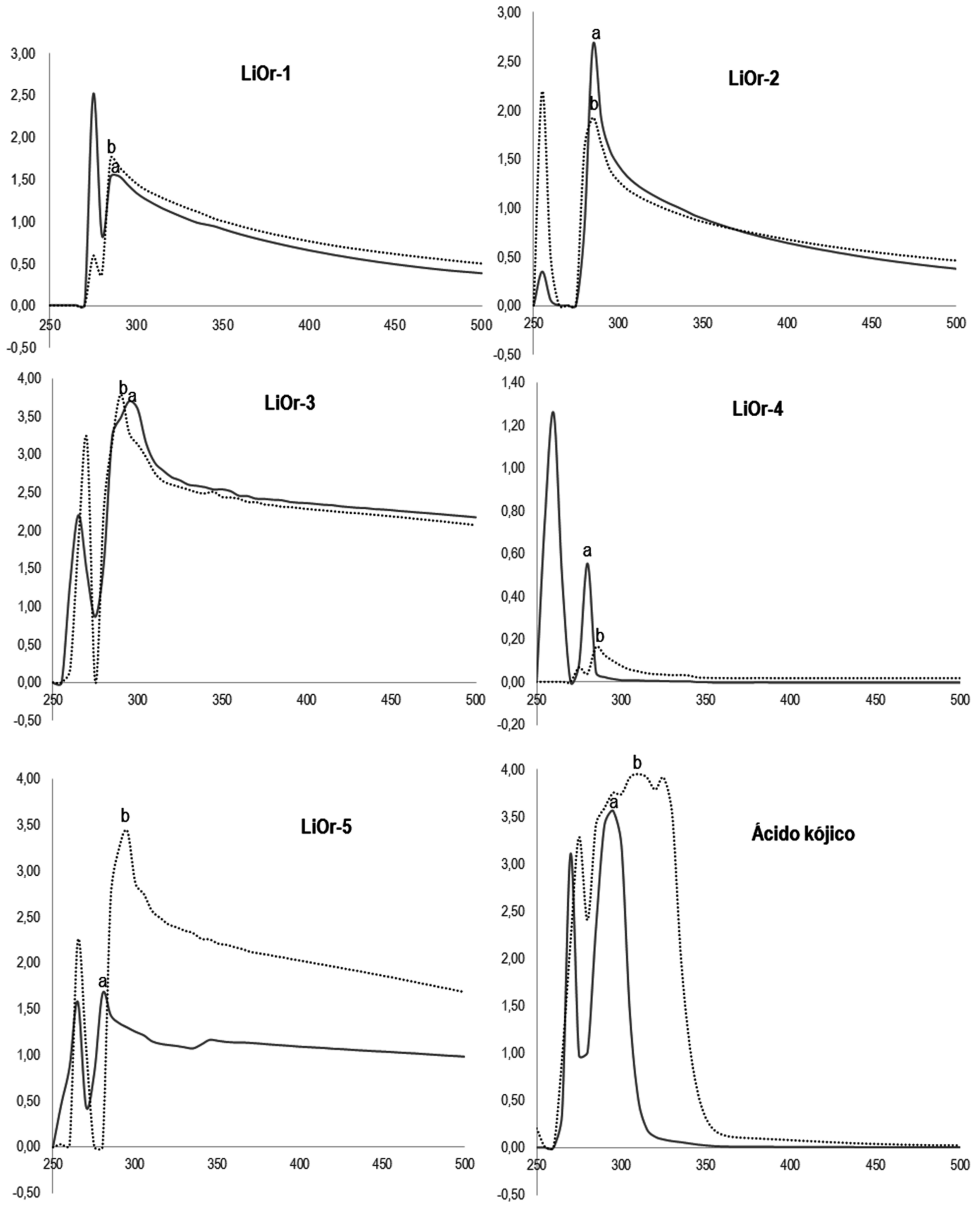
UV- vis spectrum of *L*. *origanoides* oils (1 mg.mL^-1^). (A) Without CuSO_4_ (250 μM). (B) with CuSO_4_ (250 μM).

Kojic acid caused a bathochromic shift of 15 nm (295 → 310) as well as the oil **LiOr-5** (280 → 295). The sample **LiOr-4** (260 → 285) showed a shift greater than that observed for kojic acid, about 25 to 35 nm and **LiOr-1** a shift smaller (275 → 285). However, the oils **LiOr-2** (285 → 255) and **LiOr-3** (295 → 290) promoted hypsochromic displacement, *i*.*e*., UV-Visible spectrum shift to a shorter wavelength. The results of the interaction of the samples, kojic acid and copper II ions are shown in the Fig 4.

The bathochromic behavior was observed with interactions between copper II and the samples **LiOr-1**, **LiOr-4** and **LiOr-5**. Bathochromic shift probably indicates complexation of the copper ion and the inhibitory activity of tyrosinase of the oils can be explained by the complexation of copper ions from the catalytic site of the enzyme [25,41]. The inhibition promoted by kojic acid, quercetin and kaempferol is well established from their copper chelation capacity of tyrosinase [42,43].

### Molecular docking

**Fig 5:**
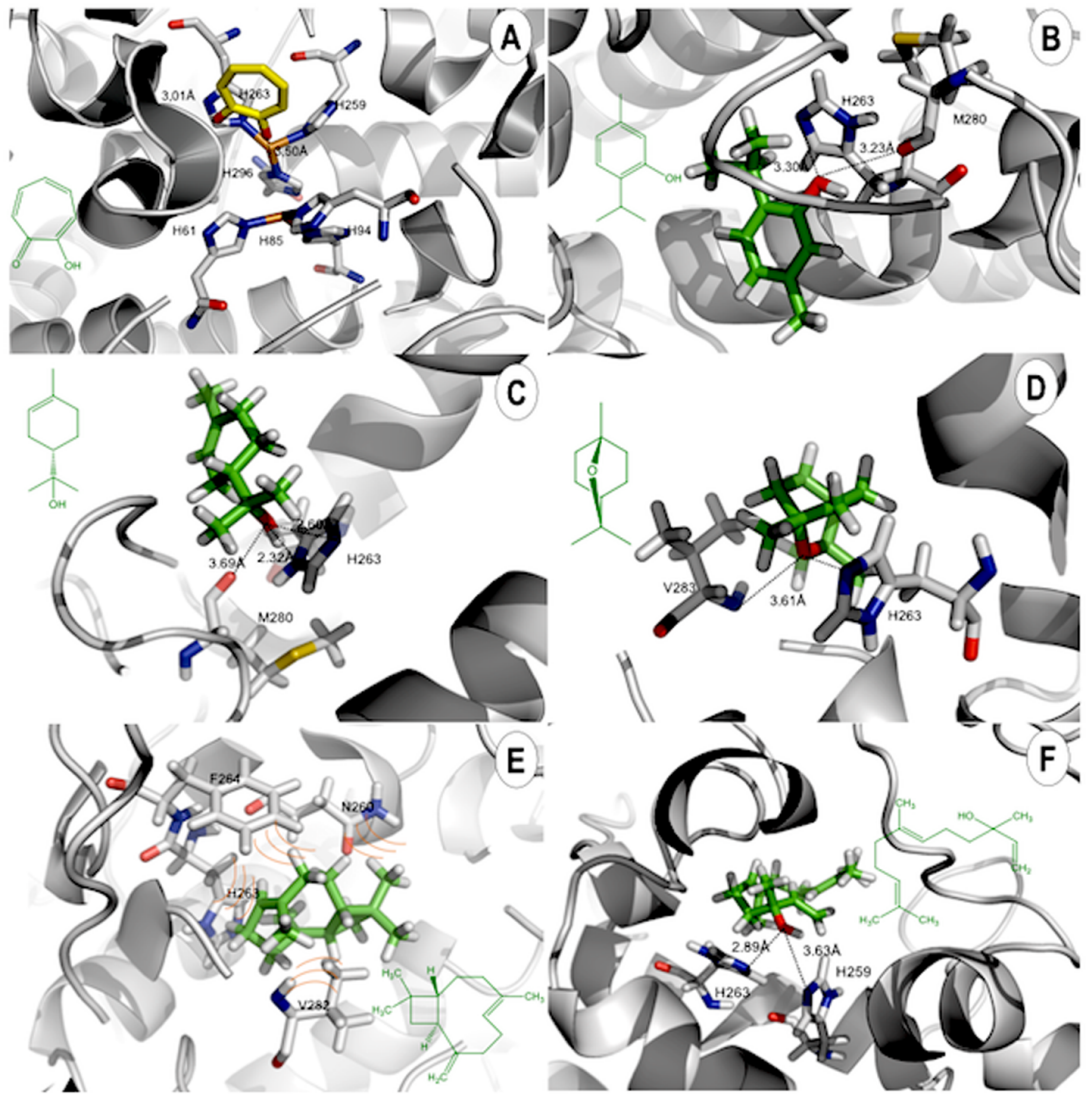
Lowest-energy docked poses of essential oil components in the binding site of tyrosinase. (A) Tropolone (yellow). (B) Thymol (green). (C) α-Terpineol (green). (D) 1,8-Cineole (green). (E) β-Caryophyllene (green). (F) (*E*)-Nerolidol (green). Key amino acid residues are shown in CPK colors. Hydrogen bonds are illustrated with dashed lines.

Previous molecular docking and quantum calculations studies have been applied to elucidate the interactions occurring in tyrosinase and their inhibitors [44,45]. The lowest-energy docked poses of 1,8-cineole, thymol, α-terpineol, β-caryophyllene and (*E*)-Nerolidol in the tyrosinase binding site are shown in Fig 5B, 5C, 5D, 5E and 5F. The docked orientations displayed that all ligands were located in the hydrophobic binding pocket surrounding the binuclear copper active site. The main compounds showed the same location of the docked than tropolone, that would contribute to their tyrosinase inhibitory potency. As a test for docking accuracy, we have re-docked the co-crystallized tropolone ligand with the enzyme (PDB 2Y9X). The lowest-energy docking pose of the ligand showed it occupying the same position in the active site, but the orientation was flipped (Fig 5). The RMSD of the docked ligand compared to the crystallized ligand was 3.138 Å (supporting information).

In this study, all docked ligands were found to interact between an oxygen atom of the ligands and Cu or histidine residue within 4 Å. In the binding pocket, common protein-ligand interactions were formed between all docked ligands and Asn260, Phe264, Ser281 and Val283 (Fig 5). The specific H-bonding interaction with Met281 was only found in the docked conformation of thymol. In order to explain the binding of these compounds, the H-bonding interactions with the other surrounding residues in the hydrophobic binding pocket were also investigated.

In Fig 5B, strong H-bonding interactions between the hydroxyl group of thymol and an oxygen atom of Met281 were formed. H-bonding interactions were also formed with His61, His85, His94, His259, His263 and His296 that are important residues coordinated with the two copper ions in the active site. The result shows strong interaction of the ligand with the residue of His263 of the catalytic site. The docked α-terpineol and (*E*)-nerolidol are shown in Fig 5D and 5F. There are additional similar H-bonding interactions with His263 presenting a distance of 2.32 Å and 2.89 Å, respectively. In the case of docked 1,8-cineole, H-bond with Val283 was also formed but this H-bonding interaction was not found with α-terpineol, β-caryophyllene or (*E*)-nerolidol. In order to explain the binding of these compounds, the H-bonding interactions with the other surrounding residues in the hydrophobic binding pocket were also investigated. In the case of β-caryophyllene only hydrophobic interactions in the catalytic site were evident. Thymol and (*E*)-nerolidol displayed comparable docking energies as kojic acid and tropolone (see Table 4).

**Table 4:** Energy calculations by MVD/MolDock Score and H-bond docking parameters.

Compounds	MolDock (Kcal∙mol^-1^)	Interaction	Residue	Distance (Å)	Atom—position
α-Terpineol	-50.3	H-bonding	His_263_(C = N-C)	2.32	O—1
			His_263_(C-N-C)	2.60	O—1
			Met_280_(O)	3.69	O—1
β-Caryophyllene	-67.3	Van der Waals	NI*		
(*E*)-Nerolidol	-79.8	H-bonding	His_259_(C-N-C)	3.63	O—1
			His_263_(C-N-C)	2.89	O—1
Thymol	-79.8	H-bonding	His_263_(C = N-C)	3.30	O—1
			Met_281_(O)	3.23	O—1
1,8-Cineole	-48.5		Val_283_(O) CIN	3.61	O—1
Kojic acid	-78.4	H-bonding	His_61_ (C-N-C)	3.10	O—2
			His_85_ (C-N-C)	3.08	O—2
			Cu (A)	2.96	O—2
Tropolone	-79.7	H-bonding	His_263_ (C-N-C)	3.01	O—2
			Cu (B)	3.50	O—2

*no H-bond interactions.

The results calculated to catalytic residues interactions showed a relationship with MolDock energy. The energy values to Thymol and (*E*)-nerolidol were -79.8 Kcal.mol^-1^. Thymol displayed two interactions with catalytic residues, which stabilizes the catalytic site and contributes to inhibitory activity (Fig 5B). (*E*)-nerolidol showed two interactions with histidine residues of catalytic triad, which contributes to the stability of ligand-protein complex (Fig 5F). The same energy values were obtained to kojic acid and tropolone -78.4 and -79.7 Kcal.mol^-1^, respectively.

### Conclusion

The presence of different chemotypes to EO of *Lippia origanoides* (LiOr) from Amazon was confirmed with predominance of monoterpenes and sesquiterpenes. The sample **LiOr-2** showed the remarked activity antioxidant and tyrosinase inhibition, which can be associated to its higher concentration of thymol. Based on the molecular docking results, the mechanism of interaction of thymol was suggested to be H-bonding interactions between Met281 and His263 residues. In the literature, there are few studies focused on tyrosinase inhibitory activity of compounds from essential oils. Based on our results, we are suggesting further studies of small phenolic compounds such as eugenol, isoeugenol, anethole, which could be promising as natural tyrosinase inhibitors.

## Supporting information

S1 Table DPPH scavenging of *Lippia origanoides* essential oils. (PDF)

S2 Table Total antioxidant capacity of *Lippia origanoides* essential oils. (PDF)

S3 Table Values of tyrosinase inhibition for *Lippia origanoides* essential oils using the substrate L-tyrosine. (PDF)

S4 Table Values of tyrosinase inhibition for *Lippia origanoides* essential oils using the substrate L-DOPA. (PDF)

S1 Fig Re-docking of tropolone (yellow) ligand and two models (pink and cyan) in the tyrosinase enzyme. (PDF)
