# Supplementary material for: Tyrosinase inhibitory activity, molecular docking studies and antioxidant potential of chemotypes of Lippia origanoides (Verbenaceae) essential oils
Source: PLoS One. 2017 May 1;12(5):e0175598. doi: 10.1371/journal.pone.0175598 (PMC5411033; doi:10.1371/journal.pone.0175598)
Supplement: S1 Table — (PDF) [file pone.0175598.s001.pdf]

S1 Table. DPPH scavenging of *Lippia origanoides* essential oils.

	Inhibition (%)				
**Experiment**	**LiOr-1**	**LiOr-2**	**LiOr-3**	**LiOr-4**	**LiOr-5**
1	40.96	77.85	27.52	18.08	49.44
2	24.40	77.20	22.64	14.98	49.76
3	17.54	77.52	25.08	16.45	49.68
Average	27.6	77.50	25.10	16.50	49.60
Standard deviation	12.0	0.30	2.40	1.50	0.20
