# Supplementary material for: Tyrosinase inhibitory activity, molecular docking studies and antioxidant potential of chemotypes of Lippia origanoides (Verbenaceae) essential oils
Source: PLoS One. 2017 May 1;12(5):e0175598. doi: 10.1371/journal.pone.0175598 (PMC5411033; doi:10.1371/journal.pone.0175598)
Supplement: S2 Table — (PDF) [file pone.0175598.s002.pdf]

S2 Table. Total antioxidant capacity of *Lippia origanoides* essential oils.

	mg ET·mL^{-1}				
**Experiment**	**LiOr-1**	**LiOr-2**	**LiOr-3**	**LiOr-4**	**LiOr-5**
1	67.34	132.63	43.56	26.84	82.35
2	38.03	131.48	34.91	21.36	82.92
3	25.89	132.06	39.23	23.96	82.77
Average	43.80	131.1	39.20	24.10	82.70
Standard deviation	21.30	0.60	4.30	2.70	0.30
