# Supplementary material for: Tyrosinase inhibitory activity, molecular docking studies and antioxidant potential of chemotypes of Lippia origanoides (Verbenaceae) essential oils
Source: PLoS One. 2017 May 1;12(5):e0175598. doi: 10.1371/journal.pone.0175598 (PMC5411033; doi:10.1371/journal.pone.0175598)
Supplement: S3 Table — (PDF) [file pone.0175598.s003.pdf]

**S3 Table. Values of tyrosinase inhibition for *Lippia origanoides* essential oils using the substrate L-tyrosine.**

| | Samples inhibition (%) | | | | | |
|---|---|---|---|---|---|---|
| **Experiment** | **Kojic acid** | **LiOr-1** | **LiOr-2** | **LiOr-3** | **LiOr-4** | **LiOr-5** |
| 1 | 75.36 | 33.92 | 84.71 | 30.59 | 66.67 | 12.31 |
| 2 | 65.94 | 42.06 | 85.19 | 48.15 | 59.41 | 12.31 |
| 3 | 78.99 | 35.29 | 84.34 | 30.59 | 61.62 | 5.61 |
| Average | 73.43 | 37.09 | 84.75 | 36.44 | 62.57 | 10.08 |
| Standard deviation | 5.50 | 3.56 | 0.35 | 8.28 | 3.04 | 3.16 |
