# Supplementary material for: Tyrosinase inhibitory activity, molecular docking studies and antioxidant potential of chemotypes of Lippia origanoides (Verbenaceae) essential oils
Source: PLoS One. 2017 May 1;12(5):e0175598. doi: 10.1371/journal.pone.0175598 (PMC5411033; doi:10.1371/journal.pone.0175598)
Supplement: S4 Table — (PDF) [file pone.0175598.s004.pdf]

S4 Table. Values of tyrosinase inhibition for *Lippia origanoides* essential oils using the substrate L-DOPA.

Experiment	Samples inhibition (%)					
	**Kojic acid**	**LiOr-1**	**LiOr-2**	**LiOr-3**	**LiOr-4**	**LiOr-5**
1	94.34	6.25	0	11.11	12.50	5.56
2	92.95	8.67	0	17.27	5.76	6.47
3	90.85	11.39	0	16.67	14.67	6.67
Average	92.71	8.77	0	15.02	10.98	6.23
Standard deviation	1.43	2.10	0	2.77	3.79	0.48
