# Supplementary material for: Tyrosinase inhibitory activity, molecular docking studies and antioxidant potential of chemotypes of Lippia origanoides (Verbenaceae) essential oils
Source: PLoS One. 2017 May 1;12(5):e0175598. doi: 10.1371/journal.pone.0175598 (PMC5411033; doi:10.1371/journal.pone.0175598)
Supplement: S1 Fig — (PDF) [file pone.0175598.s005.pdf]

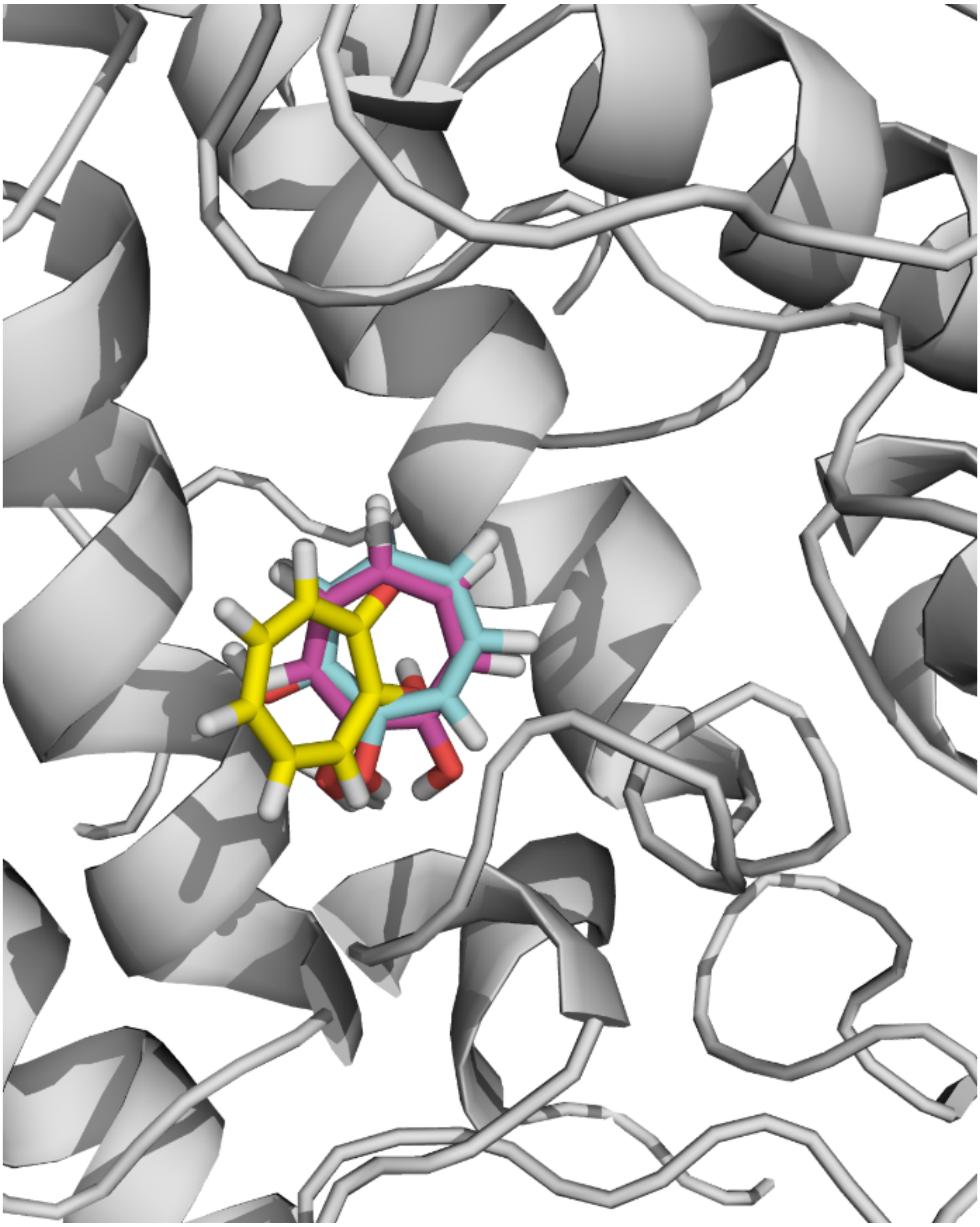

S1 Fig. Re-docking of tropolone (yellow) ligand and two models (pink and cyan) in the tyrosinase enzyme.
